# Dipeptidyl peptidase 4 expression is not associated with an activated fibroblast phenotype in idiopathic pulmonary fibrosis

**DOI:** 10.3389/fphar.2022.953771

**Published:** 2022-08-31

**Authors:** Måns Kadefors, Frida Berlin, Marie Wildt, Göran Dellgren, Sara Rolandsson Enes, Anders Aspberg, Gunilla Westergren-Thorsson

**Affiliations:** ^1^ Department of Experimental Medical Science, Lund University, Lund, Sweden; ^2^ Department of Clinical Sciences Lund, Lund University, Lund, Sweden; ^3^ Transplant Institute and Department of Cardiothoracic Surgery, Sahlgrenska University Hospital, Gothenburg, Sweden

**Keywords:** fibroblast, lung, fibrosis, idiopathic pulmonary fibrosis, dipeptidyl peptidase 4

## Abstract

Dipeptidyl peptidase 4 (DPP4) has been proposed as a marker for activated fibroblasts in fibrotic disease. We aimed to investigate whether a profibrotic DPP4 phenotype is present in lung tissue from patients with idiopathic pulmonary fibrosis (IPF). The presence of DPP4^+^ fibroblasts in normal and IPF lung tissue was investigated using flow cytometry and immunohistology. In addition, the involvement of DPP4 in fibroblast activation was examined *in vitro*, using CRISPR/Cas9 mediated genetic inactivation to generate primary DPP4 knockout lung fibroblasts. We observed a reduced frequency of primary DPP4^+^ fibroblasts in IPF tissue using flow cytometry, and an absence of DPP4^+^ fibroblasts in pathohistological features of IPF. The *in vivo* observations were supported by results *in vitro* showing a decreased expression of DPP4 on normal and IPF fibroblasts after profibrotic stimuli (transforming growth factor β) and no effect on the expression of activation markers (α-smooth muscle actin, collagen I and connective tissue growth factor) upon knockout of DPP4 in lung fibroblasts with or without activation with profibrotic stimuli.

## 1 Introduction

In idiopathic pulmonary fibrosis (IPF), progressive scarring of lung tissue obstructs the organs primary function of gas exchange which can ultimately lead to respiratory failure (Richeldi et al., 2017). Fibrosis is an outcome of dysregulated repair processes resulting in an increased activation of fibroblasts which cause an excess production and deposition of extracellular matrix. Activated fibroblasts display an increased expression of several extracellular matrix proteins, contractile proteins and growth factors, including collagens, α-smooth muscle actin and connective tissue growth factor (Hinz et al., 2007; Darby et al., 2016; Effendi and Nagano, 2022). In the case of IPF, it is believed that chronic injury to the lung epithelium triggers continued activation of surrounding fibroblasts to a pathological state involving excess extracellular matrix production and cell proliferation (Richeldi et al., 2017).

With the evolution of single cell-based technologies, our understanding of cellular heterogeneity and its extent within lung tissue has improved and previously undescribed subsets and cell states have emerged (Travaglini et al., 2020; Tsukui et al., 2020). However, the involvement and function of different fibroblast phenotypes in fibrosis still remain unclear. Identification of novel fibroblast markers, ideally accessible on the cell surface to facilitate targeting and isolation (e.g. by flow cytometry), is therefore highly warranted to design effective therapeutic interventions for IPF.

Dipeptidyl peptidase 4 (DPP4, also known as CD26) is a type II transmembrane glycoprotein with serine protease activity which is ubiquitously expressed in multiple tissues including lung (Kähne et al., 1999; Gorrell et al., 2001). Several hormones, cytokines and chemokines are included among its substrates. DPP4 also possess non-enzymatic functions including its role as a T-cell co-stimulatory protein and interactions with extracellular matrix proteins such as collagen and fibronectin, which are increased in IPF (Löter et al., 1995; Gorrell et al., 2001; Cheng et al., 2003).

In fibrotic skin conditions, as observed in systemic sclerosis and keloid, expression of DPP4 has been associated with an expanded profibrotic fibroblast phenotype (Xin et al., 2017; Soare et al., 2020). In mice, it was also reported that a fibrogenic dermal cell lineage could be identified based on DPP4 expression (Rinkevich et al., 2015). This lineage was responsible for a majority of scarring after injury (cutaneous wound), the extent of which could be reduced using a DPP4 inhibitor (diprotin A). In line with this, it was later reported that thy-1 membrane glycoprotein and DPP4 double positive (THY1^+^DPP4^+^) fibroblasts are the major producers of collagen in human skin wounds (Worthen et al., 2020). These data link DPP4 expression on fibroblasts to an activated fibroblasts phenotype which could represent a potential target in fibrosis treatment. The effect of DPP4 inhibition on fibrosis has been reported in several organs, including skin (Soare et al., 2020), heart (Hirakawa et al., 2015), kidney (Kanasaki et al., 2014), liver (Kaji et al., 2014) as well as lung (Liu and Qi, 2020; Soare et al., 2020). In a bleomycin-induced mouse model of lung fibrosis it was reported that pharmacological inhibition of DPP4 attenuated the development of lung fibrosis and reduced the expression of extracellular matrix proteins (Liu and Qi, 2020). Still, the cells directly affected by DPP4 inhibition in lung fibrosis remain unclear and the specific role of DPP4 on human lung fibroblasts in IPF has not been investigated.

Based on these findings, we hypothesized that DPP4 expression could identify a profibrotic phenotype in IPF. We have previously described subsets of endoglin and THY1 double positive (ENG^+^THY1^+^) human lung fibroblasts with high proliferative potential and observed a population of DPP4-expressing cells (Kadefors et al., 2021). In the present study, we aimed to further investigate the presence of DPP4-expressing fibroblasts in normal and end-stage IPF human lung tissue, and the potential link between DPP4 and fibroblast activation in lung fibrosis. To achieve this we used flow cytometry and immunohistology to investigate the presence of DPP4^+^ fibroblasts in patient material and studied the involvement of DPP4 in transforming growth factor β (TGF-β) induced fibroblast activation using CRISPR/Cas9 based genetic inactivation of DPP4 *in vitro*.

## 2 Materials and methods

### 2.1 Patient description and ethical approval

Lung explants from healthy donors (*n* = 11, unusable for transplantation) and patients (n = 8) with end-stage idiopathic pulmonary fibrosis (IPF) undergoing lung transplantation were acquired from Sahlgrenska University Hospital (healthy donor and IPF) and Skåne University Hospital in Lund (IPF). Donor and patient information is summarized in Supplementary Table S1. Written informed consent to participate in the study was obtained from all participants or from their closest relatives. No organs/tissue were obtained from prisoners. The study was approved by the Swedish Research Ethical Committee in Lund (FEK 2006/91) and Gothenburg (FEK 657–12/2012 and FEK 2008/413). All experimental protocols were conducted in accordance with guidelines approved by the ethical committees.

### 2.2 Isolation of primary lung fibroblasts

Primary human lung fibroblasts were isolated from parenchymal tissue using explant cultures as previously described (Hallgren et al., 2010).

### 2.3 Cell culture

Primary human lung fibroblasts were cultured in DMEM (high glucose, Gibco) supplemented with 10% Fetal Clone III (FCIII, HyClone), 1x GlutaMAX (Gibco), and either 1x antibiotic antimycotic solution (AB/AM, Sigma-Aldrich) or 0.05 mg/ml Gentamicin and 2.5 µg/ml Amphotericin B at 37°C and 8% CO_2_. Cells were passaged by detaching enzymatically with TrypLE Express (Gibco). To investigate cellular response to TGF-β1, cells were treated with recombinant human TGF-β1 protein (2 and 10 ng/ml, R&D Systems, 240-B) in DMEM with 1% FCIII and 1x GlutaMAX for 24 h or 48 h. DMEM with 1% FCIII and 1x GlutaMAX was used as a control. Cells were used in passage 4–5 (gene editing), 7–8 (TGF-β1 treatment of normal and IPF fibroblasts), 10 (phase holographic imaging) and 9–10 (TGF-β1 treatment of WT and DPP4-KO fibroblasts).

### 2.4 Isolation of lung single cell suspensions

To investigate the presence of DPP4^+^ fibroblasts in native tissue, single cell suspensions were generated from lung tissue as described. Parenchymal tissue from lung explants was dissected out by avoiding visible airways. Tissue pieces (∼30 mm^3^) were washed in PBS and further minced using scissors (<3 mm^3^ pieces). 100 U/mL DNase I (Sigma-Aldrich), 300 U/mL collagenase type I (Gibco) and 1000 U/mL hyaluronidase (Serva Electrophoresis) in PBS were used to digest the tissue for 1.5–2 h at 37°C. Digested tissue was filtered through a 100 μm filter. The cell suspension was incubated in a lysis buffer (155 mM NH_4_Cl and 10 mM KHCO_3_) for 5 min to lyse red blood cells. Cell suspensions from some donors/patients were depleted of HT2-280^+^ alveolar type 2 cells using immunomagnetic cell separation (MACS, Miltenyi). Cells were cryopreserved in culture medium mixed 1:1 (v/v) with PBS containing 15% DMSO, 50% FBS (Life Technologies) and 20 IU/ml Heparin (Leo Pharma).

### 2.5 CRISPR/Cas9 mediated gene editing

Two single gRNAs (sgRNAs, TrueGuide synthetic gRNA, Invitrogen) were designed with complementary sequences to two target DNA sequences in exon 2 of DPP4 [sgDPP4-1: A*A*C*CAC​GGG​CAC​GGU​GAU​GA, sgDPP4-2: A*G*U*CCC​AGA​AGA​ACC​UUC​CA (*modified 2′-O-Methyl bases with phosphorothioate linkages)]. Cells in passage 4-5 were transfected with TrueCut Cas9 protein v2 (Invitrogen, A36498) and sgRNA using Lipofectamine CRISPRMAX transfection reagent (Invitrogen, CMAX00003) according to manufacturer’s instructions. Briefly, for each well (12-well plate) 2 500 ng Cas9 protein, 15 pmol sgRNA and 50 uL Opti-MEM I reduced serum medium (Gibco) were mixed and 5 uL Lipofectamine Cas9 Plus Reagent was added to the mix. 3 ul Lipofectamine CRISPRMAX reagent was diluted in 50 uL Opti-MEM I medium and incubated for 1 min before mixing with the sgRNA/Cas9 ribonucleoprotein complex and incubating for 12 min to form transfection complex. 50 uL transfection complex was added to each well with 1 ml culture medium 1 day after seeding cells. Cells were incubated with transfection complex at 37 °C and 8% CO_2_ for 2 days.

### 2.6 Flow cytometry and cell sorting

**FIGURE 1:**
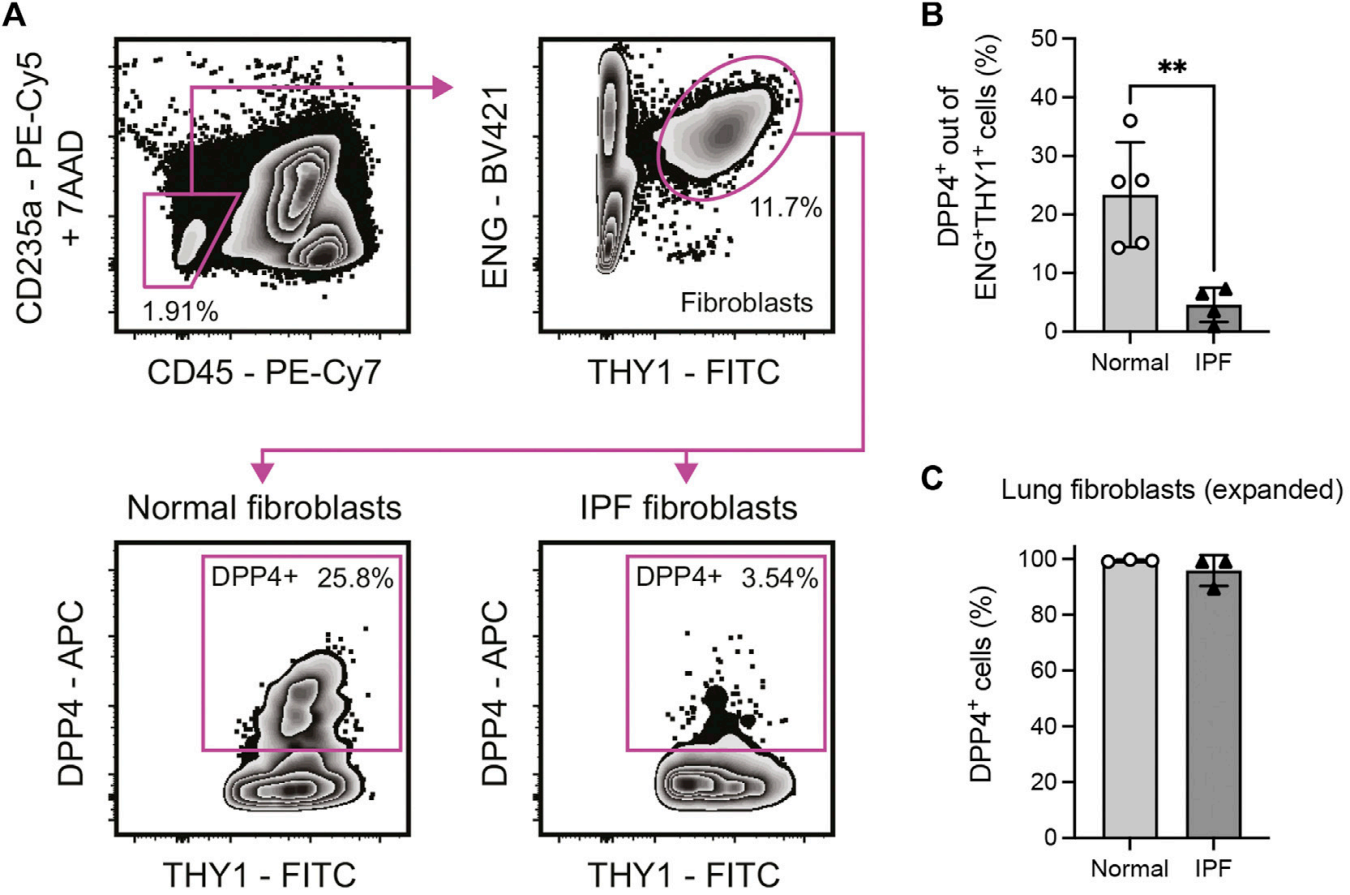
Identification of a DPP4^+^ fibroblast subset in normal and fibrotic (IPF) human lung tissue. **(A)** Flow cytometry gating of 7AAD^−^CD235a^−^CD45^−^ENG^+^THY1^+^DPP4^+^ lung fibroblasts in enzymatically dissociated single cell suspension from human lung tissue. **(B)** Percentage DPP4^+^ cells out of all CD235a^−^CD45^−^ENG^+^THY1^+^ cells in normal (*n* = 5) and IPF (*n* = 4) lung tissue determined by flow cytometry analysis. **(C)** Percentage DPP4^+^ cells in fibroblast cultures (passage 7–8) from normal (*n* = 3) and IPF (*n* = 3) lung tissue, as determined by flow cytometry analysis. No statistically significant difference was observed between culture expanded normal and IPF fibroblasts. Bars represent mean (SD). For statistical analysis, a two-tailed t-test was used. *p < 0.05, **p < 0.01.

Cells were incubated in PBS containing 3.3 mg/ml human immunoglobulin (Gammanorm, Octapharma) and 0.1% FCS (Gibco) for 10 min at 4 °C, to block non-specific binding to Fc-receptors, followed by staining with directly conjugated monoclonal antibodies (Supplementary Table S2) for 20 min at 4°C. For cryopreserved uncultured single cell suspensions, 1 mM MgCl_2_ and 100 Kunitz units/mL DNase I (Sigma-Aldrich) was added to buffers during blocking and staining. Prior to analysis, uncultured single cell suspensions were stained with 7-aminoactinomycin D (7AAD, eBioscience). All cells were filtered through a 35 µm cell strainer before analysis and/or sorting. Cells were analyzed on a BD LSR II flow cytometer or analyzed/sorted on a BD FACS Aria IIu cell sorter (BD Biosciences). Sorted cells were collected in 1.5 ml microcentrifuge tubes with 500 ul cold DMEM with 10% FCIII, 1x GlutaMAX and 1x AB/AM. Flow cytometry data was analyzed using FlowJo software version 10.8.1 (BD Bioscience). Gates for multicolor experiments in Figure 1A were set based on corresponding fluorescence-minus-one controls (Supplementary Figure S1).

### 2.7 Sequential same slide multiplex immunofluorescence and H&E staining

Formalin-fixed paraffin-embedded distal lung tissue from healthy donors (n = 3) and IPF patients (n = 3) were sectioned into 4 µm thick sections and placed on Superfrost Plus glass slides (Epredia). Slides with tissue sections were heated at 60°C for 30 min, followed by deparaffinization and rehydration. Heat-induced epitope-retrieval (HIER) was performed on the PT Tissue Link system (Dako) using Tris/EDTA buffer (pH 9.0). Sections were incubated with primary antibodies against PECAM1/CD31 (1:200, clone JC70A, Dako, M0823), DPP4/CD26 (1:200, polyclonal, R&D Systems, AF1180) and THY1/CD90 (1:200, clone EPR3132, Abcam, ab92574) or against DPP4/CD26 and pan-cytokeratin (1:100, clone AE1/AE3, Abcam, ab27988) diluted in PBS with 2% BSA at 4 °C overnight or at 37 °C for 1 h in a humid chamber. Sections were then incubated with secondary antibodies [Alexa Flour 488-conjugated F (ab')2-goat anti-mouse (Invitrogen, A11017), Alexa Flour 555-conjugated donkey anti-goat (Invitrogen, A21432) and Alexa Flour 647-conjugated chicken anti-rabbit (Invitrogen, A21443), or Alexa Flour 555-conjugated donkey anti-mouse (Invitrogen, A31570) and Alexa Flour 647-conjugated donkey anti-goat (Invitrogen, A21447)] diluted 1:200 in PBS with 1 μg/ml DAPI at room temperature for 45 min. Slides were mounted with Dako Fluorescence mounting medium (Dako) and #1 thickness cover glass. After immunofluorescence stained sections were imaged, slides were processed for histological stains to visualize tissue sections. Slides were placed in PBS for 1 h to dissolve mounting media and cover glass was removed. Slides were stained with Mayer’s hematoxylin and eosin (H&E) followed by dehydration and mounting with Pertex mounting medium (Histolab) and thickness #1 cover glass. Images were obtained with the VS120 virtual microscopy slide scanning system (Olympus). Images of whole sections were analyzed in QuPath (Bankhead et al., 2017) version 0.3.2 and regions of interest were exported to Fiji (Schindelin et al., 2012) with ImageJ version 2.0.0-rc-69/1.53j to generate figures. Quantification of stained pixel area was performed in ImageJ using the Image Calculator function. A threshold for positive staining was set for each channel (CD31, DPP4 and THY1) to generate masks of single positive pixels. CD31 positive pixels were subtracted from DPP4 and THY1 masks to generate masks of CD31 negative staining. Finally, DPP4 and THY1 double positive pixels were masked by combining the overlap pixels from single stain masks for DPP4 and THY1.

### 2.8 Live cell imaging using phase holographic imaging

Cells plated at a low concentration (1300 cells/cm^2^) in 6-well plates (TC 6 well plate, Sarstedt) were imaged using a Holomonitor M4 live cell imaging system (Phase Holographic Imaging, PHI) placed in an incubator set at 37 °C and 5% CO_2_. Images from 5-8 regions per well were captured every 15 min over 72 h. Analysis of time-lapse images was performed using HStudio (PHI). To assess cell proliferation, 3-6 regions per sample (well) were used for analysis. Fold changes of cell number at different timepoints compared to 0 h for each region were calculated. The average of fold changes across each sample and timepoint were then calculated. To assess cell motility, 7–12 cells collected from 2-3 regions for each sample were tracked and analyzed over 12 h. To assess cell morphology, 5–16 cells per sample were analyzed at the 12 h time point. Elongation was calculated as the ratio between box length and box breadth of cells.

### 2.9 RNA isolation

Cells were washed with PBS and lysed using RLT buffer (Qiagen) with 1% β-mercaptoethanol. Cell lysate was harvested using a cell scraper and homogenized by passing the lysate through a syringe. Processed cell lysate was stored in -80 °C before RNA extraction using RNeasy mini kit (Qiagen) according to the manufacturer’s instructions.

### 2.10 Quantitative real-time PCR

For cDNA synthesis QuantiTect reverse transcription kit (Qiagen) was used according to the manufacturer’s instructions, with 500 ng RNA as input. Quantitative real-time PCR was performed using QuantiFast SYBR Green PCR kit (Qiagen) together with validated QuantiTect Primer Assay probes (Qiagen) for ACTA2, COL1A1, CTGF and PPIA (see Supplementary Table S3 for information on primers used) on a StepOnePlus Real-Time PCR System (Applied Biosystems). Data was analyzed using the StepOne Software version 2.3 (Applied Biosystems). C_T_ values for each target gene were normalized (ΔC_T_) by subtracting C_T_ values of the housekeeping gene PPIA. Relative mRNA expression (fold change) compared to unstimulated WT controls for each donor and target gene combination were calculated according the 2^−ΔΔCT^ formula.

### 2.11 Statistical analysis

Data is presented as mean (SD). Individual data points shown represent biological replicates. Differences between two groups were assessed using two-tailed Student’s *t* test, and differences between multiple groups were assessed by mixed-effects analysis with Tukey’s or Šídák’s multiple comparisons test. A value below 0.05 was considered significant. Statistical analysis was performed using GraphPad Prism version 9.3.1.

## 3 Results

### 3.1 DPP4-expressing fibroblasts are decreased in IPF lungs

To evaluate expression of DPP4 on human lung fibroblast we performed flow cytometry experiments to analyze DPP4 expression on primary CD235a^−^CD45^−^THY1^+^(ENG^+^) fibroblasts isolated from lung explants (normal donors and IPF, Figure 1A). The analysis revealed the existence of a population of THY1^+^DPP4^+^ fibroblasts comprising on average 23.4% (mean, range 14.3–36.0) of THY1^+^ fibroblasts in normal lung. Unexpectedly, this fraction was significantly reduced (mean 4.6%, range 0.9–7.3) in tissue from IPF patients (Figure 1B). In contrast, close to 100% of *in vitro* expanded lung fibroblasts from both normal donors and IPF patients expressed DPP4 (Figure 1C).

### 3.2 THY1^+^DPP4^+^ cells are identified in lung vascular adventitia

**FIGURE 2:**
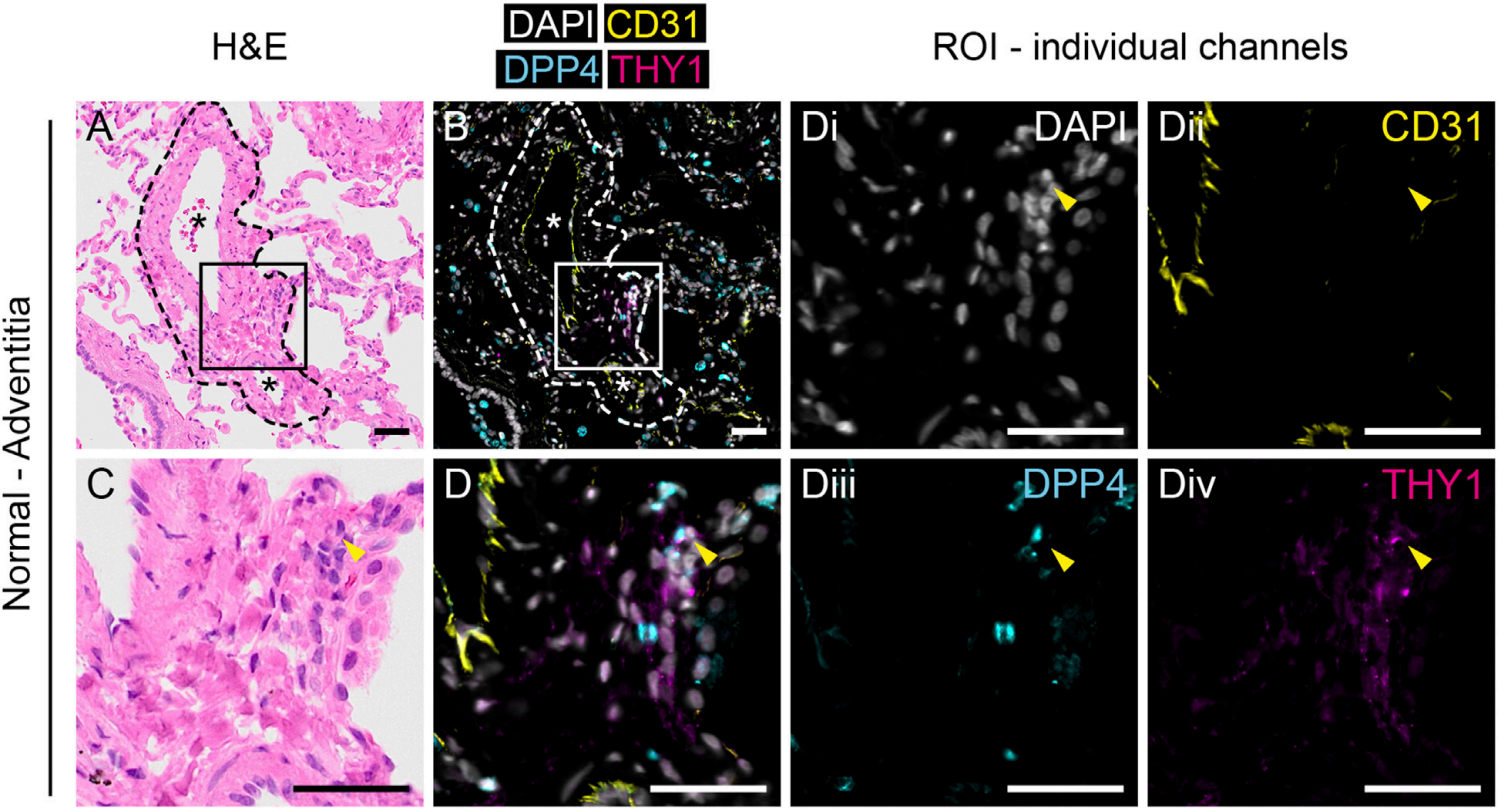
Anatomical identification of THY1^+^DPP4^+^ fibroblasts in human lung tissue. H&E **(A,C)** and immunofluorescence staining for DAPI (white), CD31 (yellow), DPP4 (cyan) and THY1 (magenta, **(B,D)** in normal human lung tissue. Region of interest (ROI) marked by rectangles (150 µm × 150 µm) in **(A, B)** are enlarged in **(C,D)**, respectively. Individual channels with DAPI (i), CD31 (ii), DPP4 (iii) and THY1 (iv) from **(D)** are shown. Arrowheads indicate CD31-THY1^+^DPP4^+^ cells localized in the tunica adventitia of pulmonary vessels. Dashed lines in **(A)** and **(B)** highlight the border between tunica adventitia and surrounding alveoli. Blood vessels are indicated with an asterisk (*). Scale bars represent 50 µm.

To investigate the localization of THY1^+^DPP4^+^ fibroblasts in lung tissue we performed multiplex immunofluorescence staining for THY1, DPP4 and CD31 together with sequential same-slide hematoxylin and eosin staining of human lung tissue. CD31 was included to visualize CD31^+^ endothelial cells. In normal lung tissue, CD31^−^THY1^+^ putative fibroblasts were mainly found in the tunica adventitia of blood vessels (Figure 2 and Supplementary Figure S2) and/or as pericytes surrounding microvasculature (data not shown). A few CD31^−^THY1^+^ fibroblasts in the tunica adventitia of vessels were positive for DPP4, consistent with the flow cytometry analysis which demonstrated that DPP4^+^ fibroblasts constitute a minority of THY1^+^ fibroblasts in human lung (Figure 2D and Supplementary Figure S2D). DPP4 expression was also observed in airway epithelium, endothelium, alveolar macrophages and unidentified CD31^−^THY1^-^ cells (including small lymphocyte-like cells, data not shown). Variable expression of DPP4 in airway epithelium was confirmed by co-stainings for DPP4 and pan-cytokeratin (Supplementary Figure S4A). We could also observe some DPP4^+^ alveolar epithelial cells (Supplementary Figure S4B).

### 3.3 THY1^+^DPP4^+^ cells are not present in histopathological features of IPF

**FIGURE 3:**
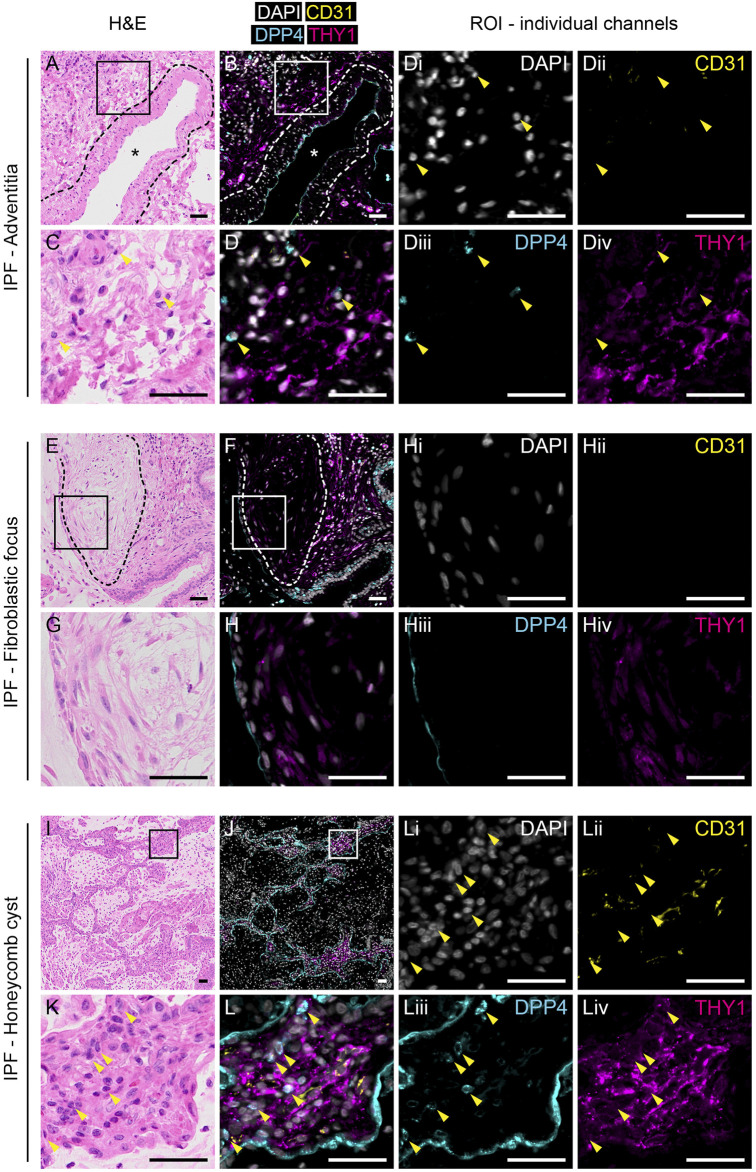
Expression of THY1 and DPP4 in IPF tissue. H&E (left panels) and immunofluorescence staining for DAPI (white), CD31 (yellow), DPP4 (cyan) and THY1 (magenta, middle and right panels) in tunica adventitia **(A–D)**, fibrotic focus **(E–H)** and honeycomb cysts **(I–L)** from IPF lung tissue. Regions of interest (ROI) marked by rectangles (150 µm × 150 µm) in **(A)**, **(B)**, **(E)**, **(F)**, **(I)** and **(J)** are enlarged in **(C)**, **(D)**, **(G)**, **(H)**, **(K)** and **(L)**, respectively. Individual channels with DAPI (i), CD31 (ii), DPP4 (iii) and THY1 (iv) from **(D)**, **(H)** and **(L)** are shown (third and fourth column). Arrowheads indicate CD31-THY1^−^DPP4^+^ cells localized to regions of CD31-THY1^+^DPP4^-^ cells. Blood vessels are indicated with an asterisk (*). Dashed lines in **(A)** and **(B)** highlight the border between tunica media and tunica adventitia. Dashed lines in **(E,F)** encircle a fibroblastic focus. Scale bars represent 50 µm.

In contrast to normal tissue, THY1^+^ cells appeared more frequent in IPF tissue (Figure 3 and Supplementary Figure S3A and S3B). Quantification of immunofluorescence stainings revealed a decreased percentage of DPP4^+^ staining in CD31^−^THY1^+^ stained areas in IPF compared to normal lung tissue, verifying flow cytometry measurements (Supplementary Figure S3C). We were not able to identify any CD31^−^THY1^+^DPP4^+^ cells in adventitial regions in fibrotic tissue (Figure 3D). We also examined the expression of THY1 and DPP4 in common histopathological features of IPF, including fibroblastic foci and honeycomb cysts. CD31^−^THY1^+^DPP4^+^ cells were not present in the fibroblastic foci examined (Figures 3E–H). Some CD31^−^THY1^+^DPP4^-^ cells could be identified in some of the fibroblastic foci, though more extensive THY1 staining was observed in fibrotic areas directly adjacent to fibroblastic foci. In the interstitial spaces of honeycomb cysts, areas rich in CD31^−^THY1^+^DPP4^-^ cells were observed, but lacking any CD31^−^THY1^+^DPP4^+^ cells. Small lymphocyte-like CD31^−^THY1^−^DPP4^+^ cells could be found within interstitial tissue next to CD31^−^THY1^+^DPP4^-^ cells (Figures 3D, 3L). We also noticed DPP4 expression in remodeled IPF epithelium. To confirm this, DPP4 and pan-cytokeratin co-stainings in IPF tissue were performed (Supplementary Figure S4). The co-stainings confirmed that some pan-cytokeratin positive epithelial cells in honeycomb cysts expressed DPP4 (Supplementary Figure S4C and S4D). However the expression appeared highly variable within and between honeycomb cysts. Taken together, these observations support the notion that a DPP4 expressing fibroblast phenotype is not expanded or does not evolve in end-stage IPF.

### 3.4 Profibrotic stimuli reduce DPP4 expression on lung fibroblasts *in vitro*

**FIGURE 4:**
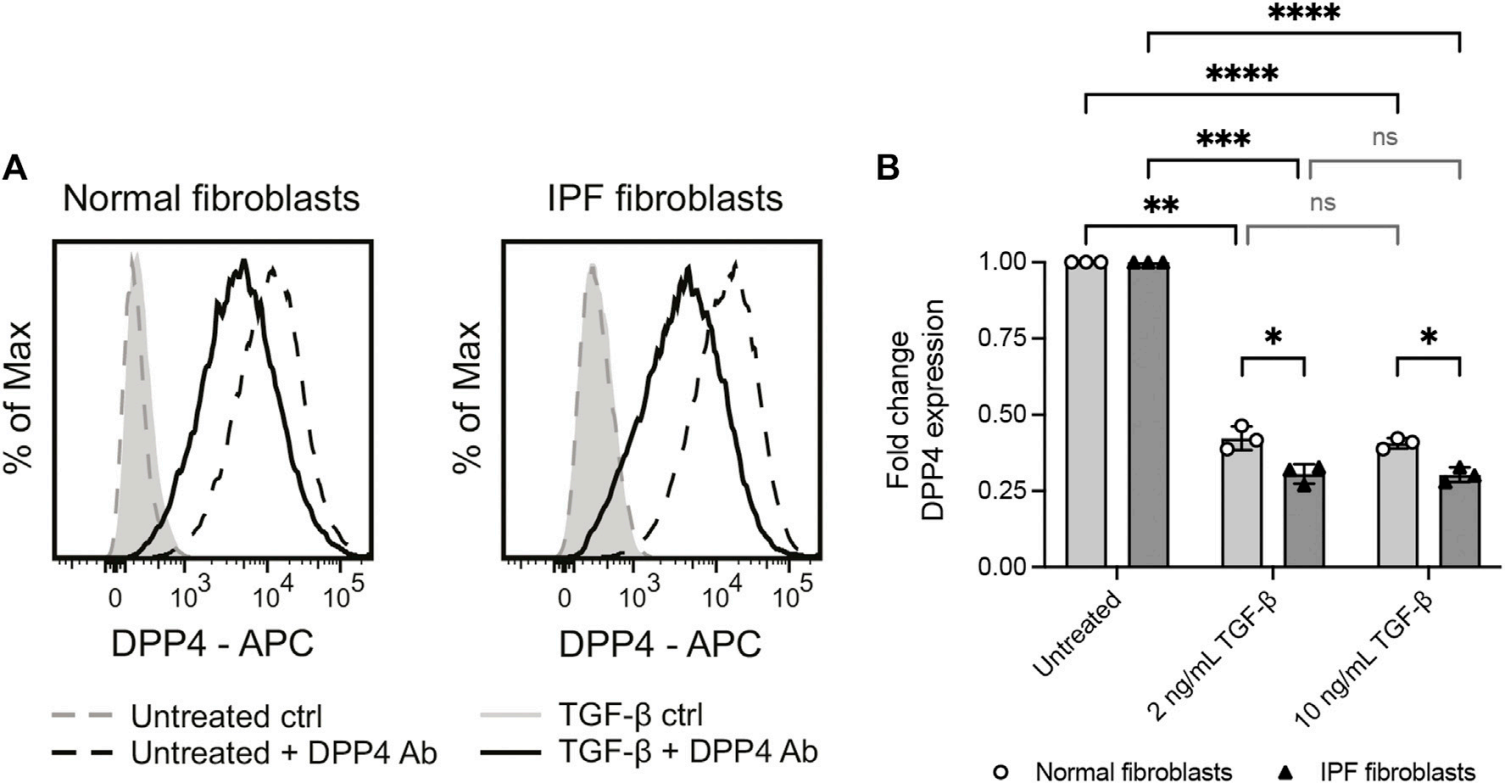
Profibrotic stimuli (TGF-β1) reduce the levels of cell surface DPP4 on normal and IPF fibroblasts. **(A)** Histograms show expression of DPP4 measured by flow cytometry on cultured normal and IPF lung fibroblasts after 48 h with or without TGF-β1 (2 ng/ml) supplemented media. Plots show data after forward/side scatter gating and doublet discrimination. **(B)** Fold change DPP4 expression in lung fibroblasts from normal (*n* = 3) or IPF (*n* = 3) tissue treated with 2 ng/ml or 10 ng/ml TGF-β1 for 48 h compared to untreated controls as measured by flow cytometry. Fold changes (normal untreated vs normal treated; IPF untreated vs IPF treated) are calculated from delta median fluorescence intensity between stained and unstained cells. Bars represent mean (SD). For statistical analysis, a mixed-effects analysis with Tukey’s (within disease groups) and Šídák’s (between disease groups) multiple comparisons test was used. *p < 0.05, **p < 0.01, ***p < 0.001, ****p < 0.0001.

It has previously been reported that myofibroblast activation of human dermal fibroblasts by TGF-β is associated with an upregulation of DPP4 (Soare et al., 2020). Yet, our observations show that DPP4 expressing fibroblasts are not more frequent in fibrotic lung tissue. To explore whether there is a mechanistic link between fibrotic stimuli and fibroblast expression of DPP4 in lung, we exposed lung fibroblast cultures from normal donors and IPF patients to the profibrotic factor TGF-β1 and measured the effect on DPP4 expression by flow cytometry. In line with our *in vivo* results, TGF-β1 stimulation caused a decrease in cell surface expression of DPP4 on fibroblasts from both normal donors and IPF patients (Figure 4). Expression of DPP4 was even further reduced on IPF fibroblasts compared to fibroblasts from normal lung upon TGF-β1 stimulation (Figure 4B).

### 3.5 DPP4 gene knockout in human lung fibroblasts using CRISPR/Cas9

**FIGURE 5:**
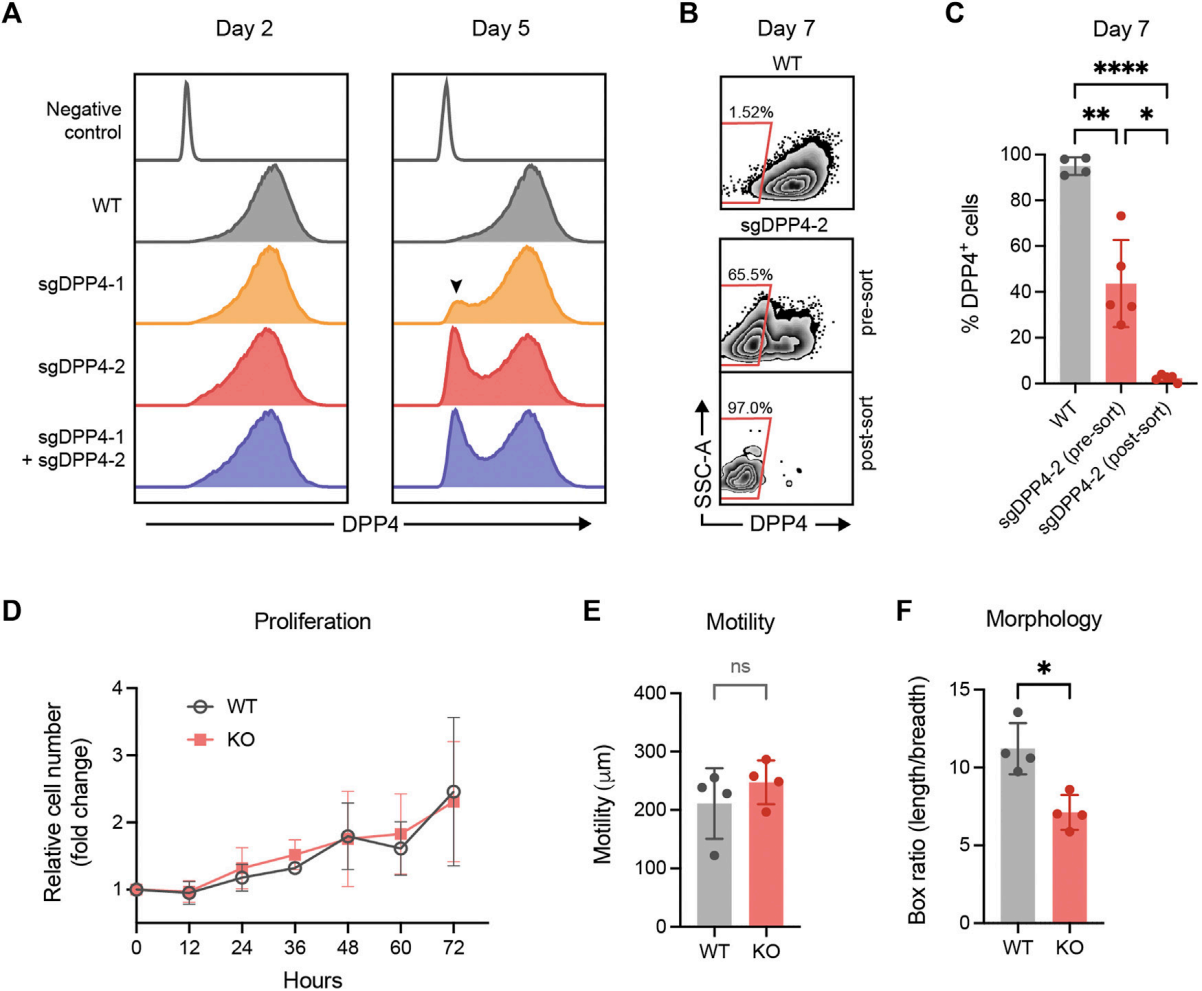
CRISPR/Cas9 mediated knockout of DPP4 in primary lung fibroblasts. **(A)** Histograms show expression of DPP4 on wild-type (WT) lung fibroblasts and lung fibroblasts after CRISPR/Cas9 mediated gene inactivation with two different sgRNAs against DPP4 and their combination. Unstained WT cells are used as a negative control (control). The peak of DPP4 knockout cells is indicated by a arrowhead. Plots show data after forward/side scatter gating and doublet discrimination. **(B)** Plots show representative flow cytometry analysis of DPP4 expression versus side scatter at day 7 on WT cells and on gene edited cells (sgDPP4-2) pre and post sorting by FACS to purify DPP4^-^ knockout cells. **(C)** Percentage DPP4^+^ cells at day 7 for WT cells (*n* = 4) and on gene edited cells (sgDPP4-2) pre and post sorting (*n* = 5) as determined by flow cytometry analysis. Lines represent mean (SD). For statistical analysis, a mixed-effects analysis with Tukey’s multiple comparisons test was used. **(D)** Cell proliferation of WT (grey circles) and DPP4-KO (red squares) cells (n = 4) over 72 h presented as cell number fold change compared to 0 h. Symbols represent mean (SD). **(E)** Cell motility over 12 h (*n* = 4). **(F)** Elongation of cells described by ratio between box length and box breadth of cells (*n* = 4). Lines represent mean (SD). For statistical analysis, a two-tailed paired t-test was used. *p < 0.05, **p < 0.01, ***p < 0.001, ****p < 0.0001.

Flow cytometry analysis revealed that all culture expanded fibroblasts expressed DPP4. This stands in contrast to the smaller fraction of DPP4^+^ cells observed in uncultured fibroblasts isolated from lung tissue, suggesting that fibroblasts alter their phenotype *in vitro* and upregulate DPP4. In order to study any potential functional role of DPP4 in fibrosis, we used CRISPR/Cas9 gene editing to knock out the gene encoding DPP4 in lung fibroblasts from normal donors. In an initial experiment, two different guide RNA (gRNA) sequences were evaluated separately and in combination. Analysis of cell surface DPP4 expression by flow cytometry was used to assess the knockout efficiency. We observed a limited effect on DPP4 expression 2 days after transfection but after 5 days a clear loss of DPP4 in a fraction of the cells could be observed. One of the gRNA sequences, sgDPP4-2, resulted in a higher knockout (KO) efficiency (sgDPP4-1: 11.8%; sgDPP4-2: 29.4%) with no apparent improvement when the two gRNAs were combined (28.2%, Figure 5A). For subsequent gene editing experiments sgDPP4-2 was used. Seven days after transfection, DPP4-KO fibroblasts were purified by flow cytometry cell sorting (Figures 5B,C). To evaluate the effect of gene editing on morphological and cell growth characteristics we performed phase holographic imaging (Sebesta et al., 2016) on live cell cultures with wild-type (WT) and DPP4-KO fibroblasts. Cell proliferation and cell motility (total distance traveled) were unaffected in DPP4-KO cells compared to WT, whereas cell elongation showed a slight reduction in DPP4-KO cells (Figures 5D–F).

### 3.6 Loss of DPP4 does not affect expression of fibrotic markers in lung fibroblasts

**FIGURE 6:**
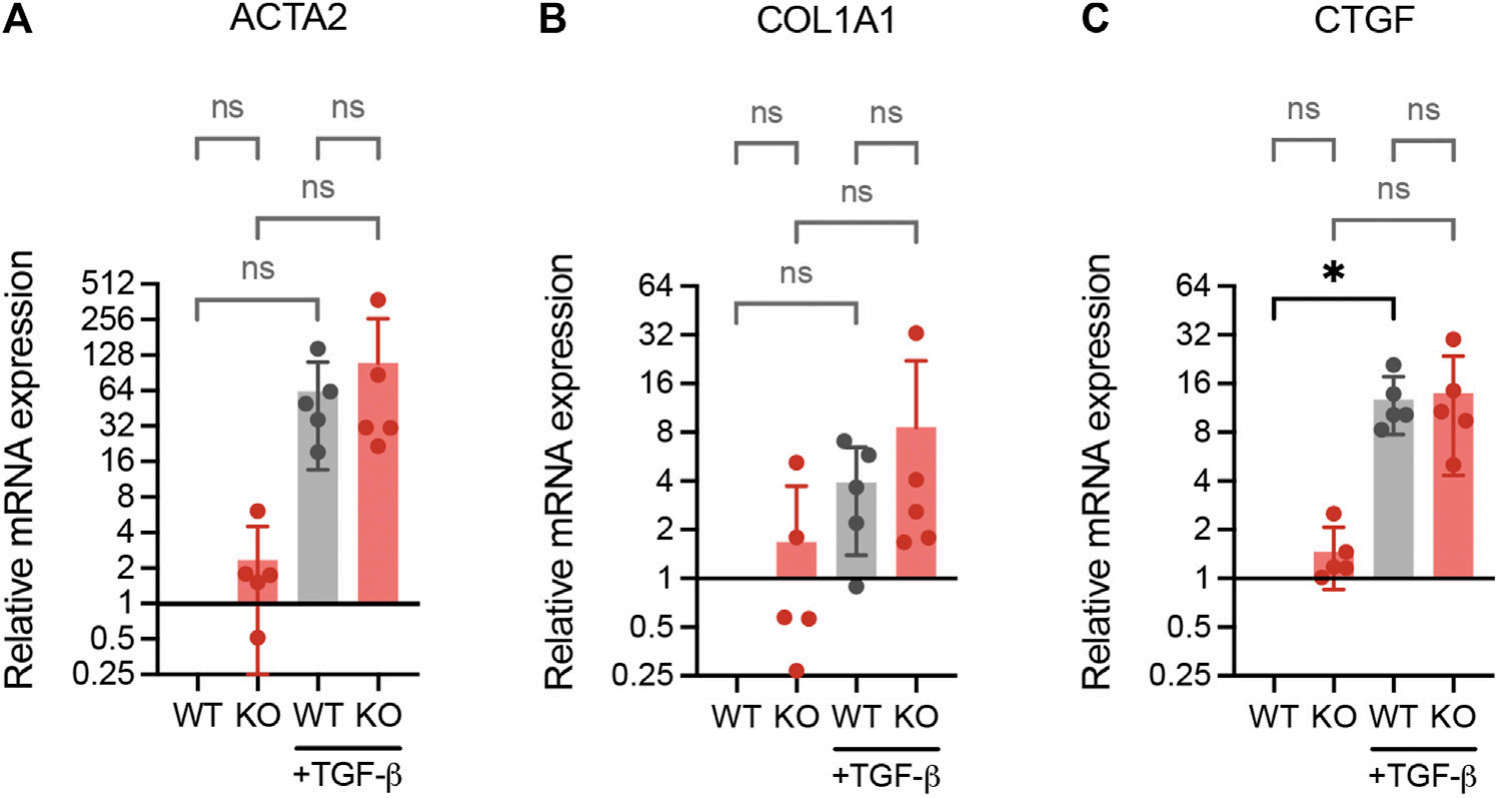
RNA expression analysis (qPCR) of fibrotic genes ACTA2 **(A)**, COL1A1 **(B)** and CTGF **(C)** in WT and DPP4-KO lung fibroblasts cultured with or without TGF-β1 (2 ng/ml) supplemented media for 24 h (*n* = 5). Data is presented as relative expression compared to unstimulated WT control. Bars represent mean. For statistical analysis, a mixed-effects analysis with Tukey’s multiple comparisons test was used. *p < 0.05.

It has been reported that inactivation of DPP4 inhibit the expression of fibrosis-related fibroblast activation markers in skin fibroblasts (Soare et al., 2020). To investigate whether DPP4 expression also influences activation markers in lung fibroblasts, we exposed WT and DPP4-KO lung fibroblasts to TGF-β1 and measured the RNA expression of α-smooth muscle actin (ACTA2), collagen I (COL1A1) and connective tissue growth factor (CTGF). ACTA2 and CTGF were upregulated upon TGF-β1 stimulation in both WT and DPP4-KO fibroblasts with no difference in the DPP4-KO compared to WT (Figures 6A–C). Basal expression levels of all three genes were similar between WT and DPP4-KO in the unstimulated controls.

## 4 Discussion

In the present study, we investigate the expression of DPP4 on fibroblasts in IPF and its involvement in human lung fibroblast activation. Based on previous findings we hypothesized that DPP4 was associated with a profibrotic phenotype. Contrary to the proposed hypothesis, the collected data suggest that DPP4 does not identify an activated profibrotic fibroblast phenotype in IPF. Our results highlight the heterogeneity of different fibrotic manifestations and the potential tissue and/or disease-specific involvement of DPP4.

In animal models, DPP4 inhibitors have been shown to reduce lung fibrosis (Suzuki et al., 2017; Liu and Qi, 2020; Soare et al., 2020). It is important to note that our results are based analysis of human tissues and cells, and could therefore differ from observations in animal models. Nevertheless, our results suggest that the therapeutic effect observed in animal models of lung fibrosis is unlikely to originate from inhibition of DPP4 on tissue-resident THY1^+^ fibroblasts. These results alone do not disqualify DPP4 as a potential target in IPF. We present data on DPP4 expression in lung fibroblasts, but DPP4 expression on other cell types may show a different involvement in IPF and thus remain a promising therapeutic avenue for IPF treatment. Interestingly, we observed expression of DPP4 on epithelial cells in remodeled lung tissue from IPF patients. As it is proposed that injury to alveolar epithelial cells contribute to the initiation of IPF, it would be of interest to investigate whether DPP4 expression on epithelial cells could have an impact on fibrosis development. Other cell types with possible expression of DPP4, such as endothelial cells and immune cells, were not interrogated in this study.

Apart for the species difference, another factor that could explain the discrepancy between our results from human lung and observed fibrotic links to DPP4 in mouse lungs is the inability of animal models to fully imitate IPF. While the tissue from end-stage IPF patients demonstrate mature scar formation from a prolonged chronic disease state, bleomycin mouse models of lung fibrosis tend to fail at recapitulating the progressive nature of IPF and instead display an initial inflammatory phase followed by a reversible fibrotic phase, which in some aspect is similar to acute lung injury (Moeller et al., 2008; Kolb et al., 2020). It is therefore possible that interventions made during the initial phase instead affect inflammatory cells causing an indirect effect on the subsequent fibrosis development. Several immune cells including T cells, B cells and NK cells express DPP4, however it is unclear what effect inhibition of DPP4 on these cells in IPF tissue would have on fibrosis (Gorrell et al., 2001). The results presented here are based on observations from lungs of end-stage IPF patients, and we do not know if the presence and involvement of DPP4^+^ fibroblasts may be different in earlier stages of disease. It is possible that DPP4^+^ fibroblast increase at an earlier disease stage but are decreased in more mature fibrotic tissue. In order to pursue DPP4 targeted therapy as an alternative to treat IPF or other fibrotic lung diseases, understanding DPP4 involvement at earlier stages of the disease would be desirable. Of relevance, McDonough and colleagues investigated the gene expression in regions with different extent of fibrosis within the IPF lung in an attempt to catch transcriptional changes at different stages of IPF (McDonough et al., 2019). In future studies it would be of interest to explore such data and similar approaches to examine DPP4 expression in IPF progression.

Our functional studies show that DPP4 expression is reduced in lung fibroblast cultures upon profibrotic stimuli with TGF-β1 and that loss of DPP4 has no impact on the expression of fibroblast activation markers. These results were unexpected as similar experiments performed on fibroblasts from skin showed the opposite response, with an increased DPP4 expression upon TGF-β stimulation and inhibited expression of activation markers upon DPP4 knockout or inhibition (Soare et al., 2020). There may be cell intrinsic mechanistic differences between fibroblasts from lung and skin tissue, which could indicate a need for tissue-specific antifibrotic treatment alternatives.

While we did not observe any impact on the expression of activation markers by DPP4 knockout, we could observe that cell elongation was reduced in fibroblasts lacking DPP4. While the mechanism behind this observation remain unknown, it indicates other functions of DPP4 on lung fibroblasts and highlight the need for additional studies on DPP4 function in lung fibroblasts.

A limitation of this study was that primary THY1^+^DPP4^-^ and THY1^+^DPP4^+^ fibroblast populations were not used for further functional studies. We observed homogeneous DPP4 expression on culture expanded lung fibroblasts suggesting that the *in vivo* phenotypes change *in vitro*. It is possible that other phenotypical difference between these populations may be involved in fibroblast activation. Instead, we investigated the specific involvement of DPP4 in lung fibroblast activation using genetic inactivation of DPP4.

Importantly, it should be mentioned that there are currently no viable markers to target all fibroblast populations. To identify and isolate fibroblasts we used THY1 which has been used previously to describe a subset of THY1^+^DPP4^+^ collagen producing fibroblasts in human skin which are increased in wound healing (Worthen et al., 2020).

In future studies, it would be of interest to investigate the role of DPP4 on other cell types in IPF. In addition, to further elucidate disease-specific differences in fibrosis, it would be of interest to compare cells from fibrotic lung tissue of different diseases, such as systemic sclerosis and acute lung injury.

In summary, we have provided evidence that DPP4-expressing fibroblasts are not involved in IPF, and as such DPP4 targeted therapy in IPF is unlikely to have a direct effect on fibroblasts. This may guide future decisions regarding the development of IPF treatments.

## Data Availability

The original contributions presented in the study are included in the article/Supplementary Material, further inquiries can be directed to the corresponding author.

## Abbreviations

IPF, Idiopathic pulmonary fibrosis; DPP4, Dipeptidyl peptidase 4; TGF-β, Transforming growth factor beta; CRISPR, Clustered regularly interspaced short palindromic repeats; sgRNA, Single guide RNA; WT, Wild-type; KO, Knockout; FACS, Fluorescence-activated cell sorting; 7AAD, 7-Aminoactinomycin D; H&E, Hematoxylin and eosin.

## References

[B1] Bankhead P. Loughrey M. B. Fernandez J. A. Dombrowski Y. McArt D. G. Dunne P. D. (2017). QuPath: Open source software for digital pathology image analysis. Sci. Rep. 7 (1), 16878–16887. Nature Publishing Group. 10.1038/s41598-017-17204-5 29203879 PMC5715110

[B2] Cheng H. C. Abdel-Ghany M. Pauli B. U. (2003). A novel consensus motif in fibronectin mediates dipeptidyl peptidase IV adhesion and metastasis. J. Biol. Chem. 278 (27), 24600–24607. Elsevier. 10.1074/jbc.M303424200 12716896

[B3] Darby I. A. Zakuan N. Billet F. Desmouliere A. (2016). The myofibroblast, a key cell in normal and pathological tissue repair. Cell. Mol. Life Sci. 73 (6), 1145–1157. 10.1007/s00018-015-2110-0 26681260 PMC11108523

[B4] Effendi W. I. Nagano T. (2022). Connective tissue growth factor in idiopathic pulmonary fibrosis: Breaking the bridge. Int. J. Mol. Sci. 23 (11), 6064. 10.3390/ijms23116064 35682743 PMC9181498

[B5] Gorrell M. D. Gysbers V. McCaughan G. W. (2001). CD26: A multifunctional integral membrane and secreted protein of activated lymphocytes. Scand. J. Immunol. 54, 249–264. John Wiley & Sons, Ltd. 10.1046/j.1365-3083.2001.00984.x 11555388

[B6] Hallgren O. Nihlberg K. Dahlback M. Bjermer L. Eriksson L. T. Erjefalt J. S. (2010). Altered fibroblast proteoglycan production in COPD. Respir. Res. 11 (1), 55. BioMed Central. 10.1186/1465-9921-11-55 20459817 PMC2886021

[B7] Hinz B. Phan S. H. Thannickal V. J. Galli A. Bochaton-Piallat M. L. Gabbiani G. (2007). The myofibroblast: One function, multiple origins. Am. J. Pathol. 170 (6), 1807–1816. 10.2353/ajpath.2007.070112 17525249 PMC1899462

[B8] Hirakawa H. Zempo H. Ogawa M. Watanabe R. Suzuki J. I. Akazawa H. (2015). A DPP-4 inhibitor suppresses fibrosis and inflammation on experimental autoimmune myocarditis in mice. PLoS ONE 10 (3), e0119360. Public Library of Science. 10.1371/journal.pone.0119360 25768281 PMC4359137

[B9] Kadefors M. Rolandsson Enes S. Ahrman E. Michalikova B. Lofdahl A. Dellgren G. (2021). CD105+CD90+CD13+ identifies a clonogenic subset of adventitial lung fibroblasts. Sci. Rep. 11 (1), 1–13. 24417. Nature Publishing Group. 10.1038/s41598-021-03963-9 34952905 PMC8709856

[B10] Kähne T. Lendeckel U. Wrenger S. Neubert K. AnSorge S. ReinholD D. (1999). Dipeptidyl peptidase IV: A cell surface peptidase involved in regulating T cell growth (review). Int. J. Mol. Med. 4 (1), 3–15. 10.3892/ijmm.4.1.3 10373631

[B11] Kaji K. Yoshiji H. Ikenaka Y. Noguchi R. Aihara Y. Douhara A. (2014). Dipeptidyl peptidase-4 inhibitor attenuates hepatic fibrosis via suppression of activated hepatic stellate cell in rats. J. Gastroenterol. 49 (3), 481–491. Springer-Verlag Tokyo. 10.1007/s00535-013-0783-4 23475323

[B12] Kanasaki K. Shi S. Kanasaki M. He J. Nagai T. Nakamura Y. (2014). Linagliptin-mediated DPP-4 inhibition ameliorates kidney fibrosis in streptozotocin-induced diabetic mice by inhibiting endothelial-to-mesenchymal transition in a therapeutic regimen. Diabetes 63 (6), 2120–2131. American Diabetes Association. 10.2337/db13-1029 24574044

[B13] Kolb P. Upagupta C. Vierhout M. Ayaub E. Bellaye P. S. Gauldie J. (2020). The importance of interventional timing in the bleomycin model of pulmonary fibrosis. Eur. Respir. J. 55 (6), 1901105. 10.1183/13993003.01105-2019 32165401

[B14] Liu Y. Qi Y. (2020). Vildagliptin, a CD26/DPP4 inhibitor, ameliorates bleomycin-induced pulmonary fibrosis via regulating the extracellular matrix. Int. Immunopharmacol. 87, 106774. Elsevier. 10.1016/j.intimp.2020.106774 32731178

[B15] Löter K. Zeilinger K. Schuppan D. Reutter W. (1995). The cysteine-rich region of dipeptidyl peptidase IV (CD 26) is the collagen binding site. Biochem. Biophys. Res. Commun. 217 (1), 341–348. Academic Press. 10.1006/bbrc.1995.2782 8526932

[B16] McDonough J. E. Ahangari F. Li Q. Jain S. Verleden S. E. Herazo-Maya J. (2019). Transcriptional regulatory model of fibrosis progression in the human lung’. JCI Insight 4 (22), 131597. American Society for Clinical Investigation. 10.1172/jci.insight.131597 31600171 PMC6948862

[B17] Moeller A. Ask K. Warburton D. Gauldie J. Kolb M. (2008). The bleomycin animal model: A useful tool to investigate treatment options for idiopathic pulmonary fibrosis? Int. J. Biochem. Cell Biol. 40, 362–382. NIH Public Access. 10.1016/j.biocel.2007.08.011 17936056 PMC2323681

[B18] Richeldi L. Collard H. R. Jones M. G. (2017). Idiopathic pulmonary fibrosis. Lancet 389, 1941–1952. Elsevier. 10.1016/S0140-6736(17)30866-8 28365056

[B19] Rinkevich Y. Walmsley G. G. Hu M. S. Maan Z. N. Newman A. M. Drukker M. (2015). Skin fibrosis. Identification and isolation of a dermal lineage with intrinsic fibrogenic potential. Sci. (New York, N.Y.) 348 (6232), aaa2151. American Association for the Advancement of Science. 10.1126/science.aaa2151 PMC5088503 25883361

[B20] Schindelin J. Arganda-Carreras I. Frise E. Kaynig V. Longair M. Pietzsch T. (2012). Fiji: An open-source platform for biological-image analysis. Nat. Methods 9, 676–682. Nature Publishing Group. 10.1038/nmeth.2019 22743772 PMC3855844

[B21] Sebesta M. Egelberg P. Langberg A. Lindskov J. H. Aim K. Janicke B., (2016). “HoloMonitor M4: Holographic imaging cytometer for real-time kinetic label-free live-cell analysis of adherent cells,” in Quantitative phase imaging II (Bellingham, Washington, United States: SPIE), 971813. 10.1117/12.2216731

[B22] Soare A. Gyorfi H. A. Matei A. E. Dees C. Rauber S. Wohlfahrt T. (2020). Dipeptidylpeptidase 4 as a marker of activated fibroblasts and a potential target for the treatment of fibrosis in systemic sclerosis. Arthritis Rheumatol. 72 (1), 137–149. John Wiley & Sons, Ltd. 10.1002/art.41058 31350829

[B23] Suzuki T. Tada Y. Gladson S. Nishimura R. Shimomura I. Karasawa S. (2017). Vildagliptin ameliorates pulmonary fibrosis in lipopolysaccharide-induced lung injury by inhibiting endothelial-to-mesenchymal transition. Respir. Res. 18 (1), 177–211. BioMed Central Ltd. 10.1186/s12931-017-0660-4 29037205 PMC5644255

[B24] Travaglini K. J. Nabhan A. N. Penland L. Sinha R. Gillich A. Sit R. V. (2020). A molecular cell atlas of the human lung from single-cell RNA sequencing. Nature 587 (7835), 619–625. Nature Research. 10.1038/s41586-020-2922-4 33208946 PMC7704697

[B25] Tsukui T. Sun K. H. Wetter J. B. Wilson-Kanamori J. R. Hazelwood L. A. Henderson N. C. (2020). Collagen-producing lung cell atlas identifies multiple subsets with distinct localization and relevance to fibrosis. Nat. Commun. 11 (1), 1920–2016. Nature Research. 10.1038/s41467-020-15647-5 32317643 PMC7174390

[B26] Worthen C. A. Cui Y. Orringer J. S. Johnson T. M. Voorhees J. J. Fisher G. J. (2020). CD26 identifies a subpopulation of fibroblasts that produce the majority of collagen during wound healing in human skin. J. Invest. Dermatol. 140 (12), 2515–2524. Elsevier B.V. 10.1016/j.jid.2020.04.010 32407715 PMC7655599

[B27] Xin Y. Wang X. Zhu M. Qu M. Bogari M. Lin L. (2017). Expansion of CD26 positive fibroblast population promotes keloid progression. Exp. Cell Res. 356 (1), 104–113. Academic Press. 10.1016/j.yexcr.2017.04.021 28454879

