# Supplementary material for: Dipeptidyl peptidase 4 expression is not associated with an activated fibroblast phenotype in idiopathic pulmonary fibrosis
Source: Front Pharmacol. 2022 Aug 31;13:953771. doi: 10.3389/fphar.2022.953771 (PMC9473336; doi:10.3389/fphar.2022.953771)

Supplementary Material

1 Supplementary Tables

Supplementary Table S1. Donor and patient tissue information for lung explant material used in the study. A = multicolor flow cytometry (primary uncultured fibroblasts), B = flow cytometry (cultured fibroblasts +/-TGF-β), C = CRISPR/Cas9 gene editing (DPP4-KO), D = immunofluorescence (FFPE), E = phase holographic imaging (WT vs KO), F = qPCR (WT vs KO, +/-TGF-β)

Donor/ patient no.	Group	Lung transplant (donor/recipient)	Lung disease	Smoking history	Sex	Age at explant (years)	HT2-280 depletion*	Used in experiment					
								A	B	C	D	E	F
N01	Control	donor	No	Never	F	46-66	-		X				
N02	Control	donor	No	Former	F	46-66	-		X	X			X
N03	Control	donor	No	Never	M	56	-		X				
N04	Control	donor	No	Former	M	62	-				X		
N05	Control	donor	No	Current	M	37	No	X					
N06	Control	donor	No	Former	M	66	Yes	X		X	X	X	X
N07	Control	donor	No	Never	M	68	Yes	X		X	X	X	X
N08	Control	donor	No	Former	M	26	Yes	X					
N09	Control	donor	No	Current	M	66	Yes	X					
N10	Control	donor	No	Never	F	65	-			X		X	X
N11	Control	donor	No	Former	M	74	-			X		X	X
F01	IPF	recipient	IPF	Never	M	59	No	X					
F02	IPF	recipient	IPF	Former	M	63	Yes	X					
F03	IPF	recipient	IPF	Former	F	57	-		X				
F04	IPF	recipient	IPF	Former	F	62	-		X				
F05	IPF	recipient	IPF	Former	M	57	-		X		X		
F06	IPF	recipient	IPF	Never	M	51	-				X		
F07	IPF	recipient	IPF	Former	F	65	Yes	X			X		
F08	IPF	recipient	IPF	Former	M	64	No	X					

*Cell suspension used for flow cytometry were depleted of HT2-280^{+} alveolar type 2 cells

Supplementary Table S2. Antibodies used for flow cytometry.

Antibody	Dilution	Clone	Company	Catalog no.
ENG/CD105-BV421	1:10	266	BD Biosciences	563920/566265
CD235a-PE-Cy5	1:100	GA-R2	BD Biosciences	559944/561776
DPP4/CD26-APC	1:20	M-A261	BD Biosciences	563670
CD45-PE-Cy7	1:100	HI30	BD Biosciences	560915
THY1/CD90-FITC	1:400	5E10	BD Biosciences	555595

Supplementary Table S3. Primers used for quantitative real-time PCR.

Primer name	Target gene	HGNC symbol	Company	Catalog no.
Hs_ACTA2_1_SG	Actin Alpha 2, Smooth Muscle	ACTA2	Qiagen	QT00088102
Hs_COL1A1_1_SG	Collagen Type I Alpha 1 Chain	COL1A1	Qiagen	QT00037793
Hs_CTGF_1_SG	Connective Tissue Growth Factor/Cellular Communication Network Factor 2	CTGF/CCN2	Qiagen	QT00052899
Hs_PPIA_1_SG	Peptidylprolyl Isomerase A	PPIA	Qiagen	QT00052311

2 Supplementary Figure Legends

Supplementary Figure S1. Fluorescence-minus-one controls (excluding antibody/dye in one channel) for multicolor flow cytometry analysis of 7AAD$^-$CD235a$^-$CD45$^-$ENG$^+$THY1$^+$DPP4$^+$ fibroblasts from normal and IPF tissue. Plots show controls from one normal donor (**A**-**E**) and one IPF patient (**F**). Data is shown after gating on single cells (**A** and **B**), single cells followed by gating on 7AAD$^-$CD235a$^-$CD45$^-$ cells (**C** and **D**) and single cells followed by gating on 7AAD$^-$CD235a$^-$CD45$^-$ENG$^+$THY1$^+$ cells (**E** and **F**).

Supplementary Figure S2. Additional images of THY1$^+$DPP4$^+$ fibroblasts in human lung tissue. H&E (**A** and **C**) and immunofluorescence staining for DAPI (white), CD31 (yellow), DPP4 (cyan) and THY1 (magenta, **B** and **D**) in normal human lung tissue. Region of interest (ROI) marked by rectangles (150 µm x 150 µm) in (**A**) and (**B**) are enlarged in (**C**) and (**D**), respectively. Individual channels with DAPI (i), CD31 (ii), DPP4 (iii) and THY1 (iv) from (**D**) are shown. Arrowheads indicate CD31-THY1$^+$DPP4$^+$ cells localized in the tunica adventitia of pulmonary vessels. Dashed lines in (**A**) and

(**B**) highlight the border between the tunica media and adventitia of a pulmonary vessel. The vessel lumen is indicated with an asterisk (*). Scale bars represent 50 µm.

Supplementary Figure S3. (**A** and **B**) Immunofluorescence staining of DAPI (white), CD31 (yellow), DPP4 (cyan) and THY1 (magenta) in normal (n=3, **A**) and IPF (n=3, **B**) tissue. Scale bars represent 1000 µm. (**C**) Quantification of the percentage of $DPP4^+$ area in the total $CD31^-THY1^+$ area in immunofluorescence stainings in (**A**) and (**B**). Bars represent mean (SD). For statistical analysis, a two-tailed t-test was used. *$P < 0.05$, **$P < 0.01$.

Supplementary Figure S4. Expression of DPP4 in lung epithelium from normal (n=3) and IPF (n=3) lungs. Immunofluorescence staining for DAPI (white), DPP4 (cyan) and pan-cytokeratin (panCK, magenta) in normal airway (**A**) and alveoli (**B**) and in honeycomb cysts of IPF tissue (**C** and **D**). Rectangles indicate regions of interest (150 µm x 150 µm) enlarged in (i), (ii) and (iii). Arrowheads indicate examples of $panCK^+DPP4^+$ cells. Scale bars represent 100 µm.

3 Supplementary Figures

Supplementary Figure S1

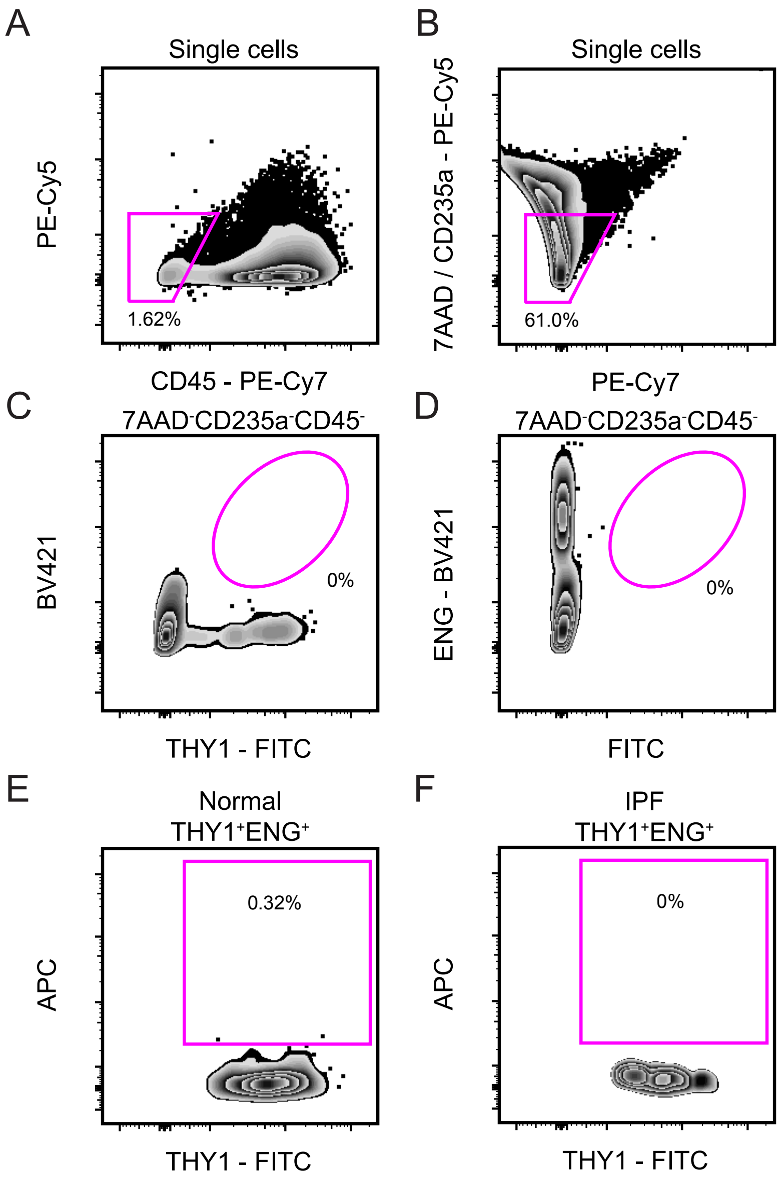

Supplementary Figure S2

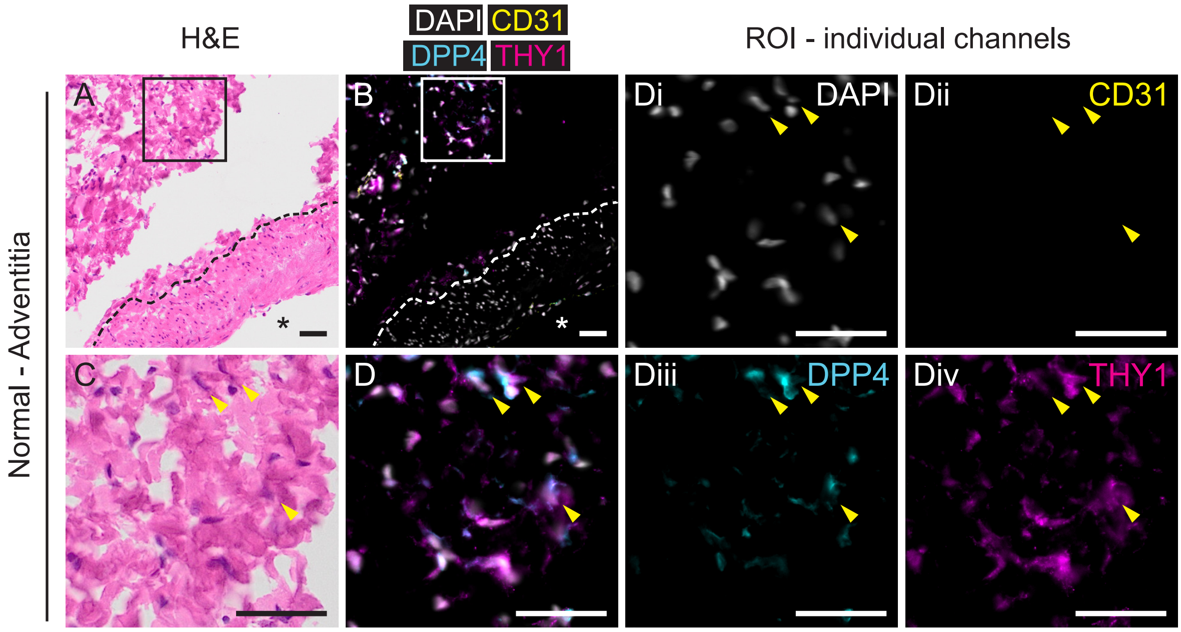

Supplementary Figure S3

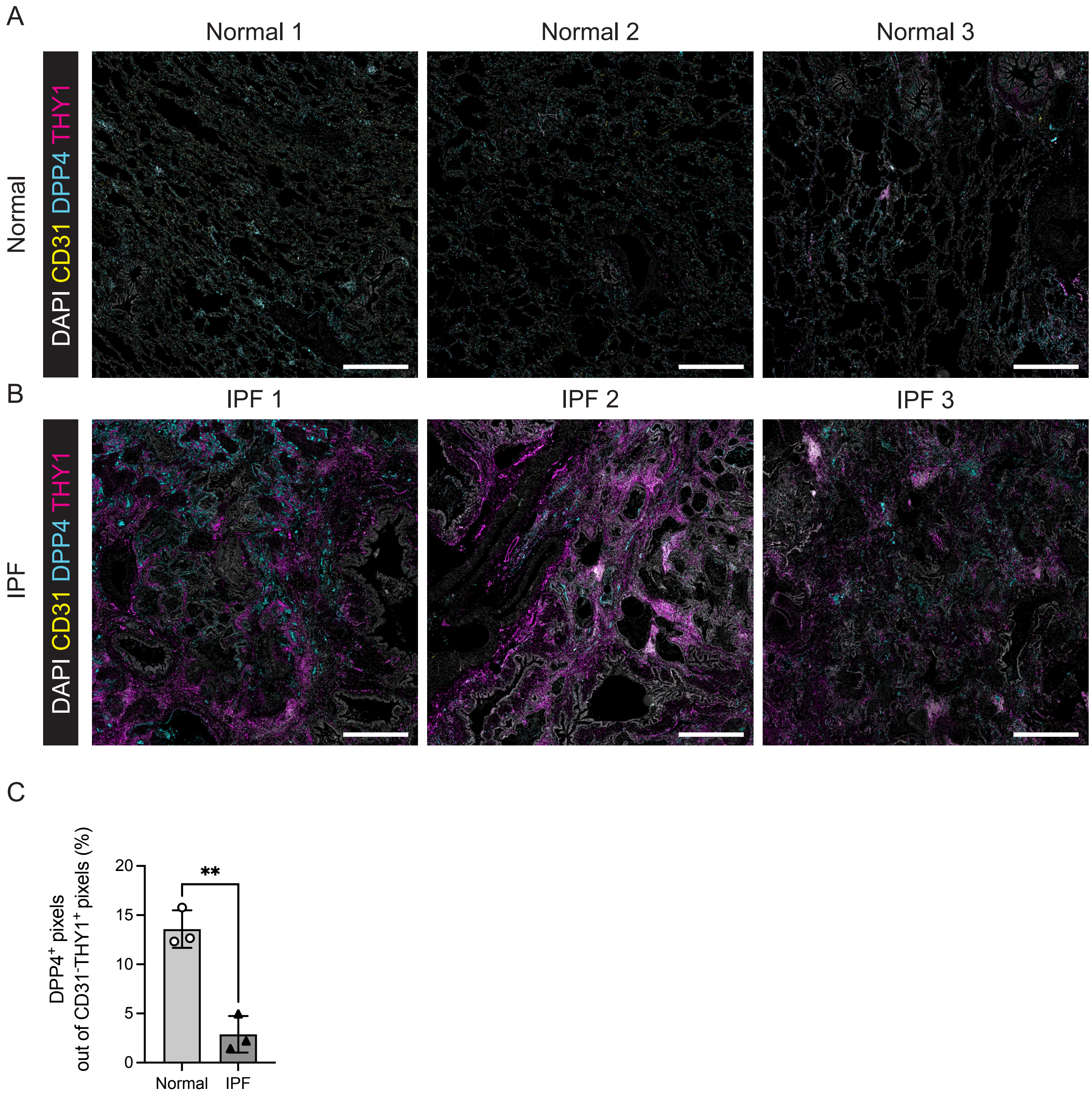

Supplementary Figure S4

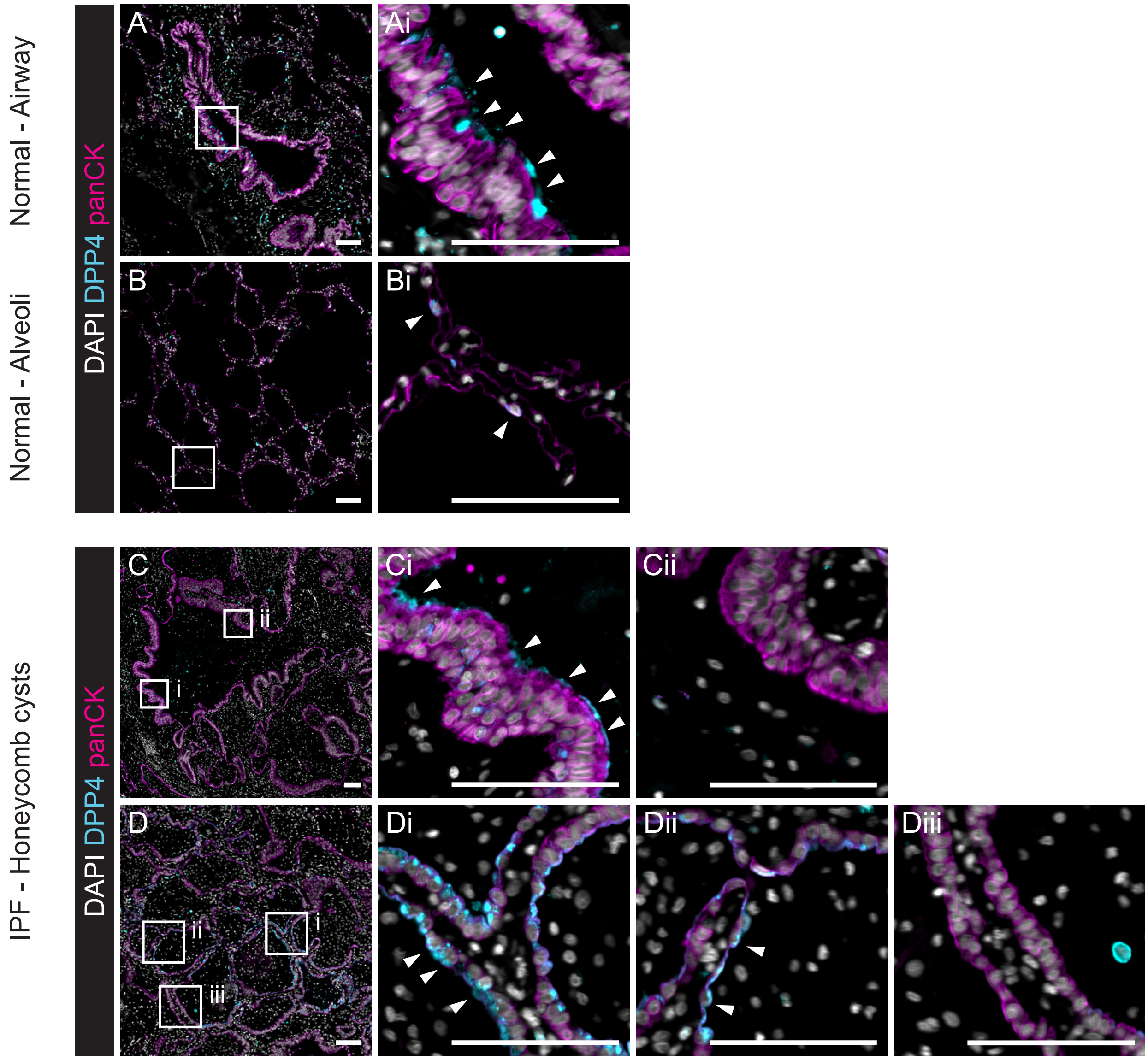

Supplement: Supplementary file 1 [file DataSheet1.pdf]
